# Diagnosis and Monitoring of Metabolic Dysfunction Associated with Fatty Liver Disease in Primary Care Patients with Risk Factors—EsteatoGal Study

**DOI:** 10.3390/jcm14093089

**Published:** 2025-04-29

**Authors:** Nerea Sánchez-Varela, Sergio Cinza-Sanjurjo, Tatiana Danif-Ferreira, Liseth I. Medina Araujo, Diego G. Mosteiro Miguéns, Daniel Rey-Aldana, Manuel Portela-Romero, on behalf of the EsteatoGal Researchers

**Affiliations:** 1 Centro de Salud Concepción Arenal, Área Sanitaria Integrada Santiago de Compostela, 15701 Santiago de Compostela, A Coruña, Spain; nerea.sanchez.varela@sergas.es (N.S.-V.); liseth.medina.araujo@sergas.es (L.I.M.A.); diego.gabriel.mosteiro.miguens@sergas.es (D.G.M.M.);; 2 Centro de Salud Milladoiro, Área Sanitaria Integrada Santiago de Compostela, 15895 O Milladoiro, A Coruña, Spain; 3 Instituto de Investigación Sanitaria de Santiago de Compostela (IDIS), 15706 Santiago de Compostela, A Coruña, Spain; daniel.rey.aldana@sergas.es; 4 Centro de Investigación Biomédica en Red de Enfermedades Cardiovasculares (CIBERCV), 28029 Madrid, Madrid, Spain; 5 Departamento de Medicina, Universidad de Santiago de Compostela (USC), 15782 Santiago de Compostela, A Coruña, Spain; 6 Centro de Salud A Estrada, Área Sanitaria Integrada Santiago de Compostela, 36680 A Estrada, Pontevedra, Spain; tatiana.danif.ferreira@sergas.es

**Keywords:** non-alcohol fatty liver disease, hepatic fibrosis, elastography, ultrasound, primary care

## Abstract

**Objective**: The objective of this study was to calculate the epidemiological impact of metabolic dysfunction associated with fatty liver disease (MAFLD) and hepatic fibrosis in primary care (PC). Secondarily, we assessed the correlation between serological markers (FIB-4, ELF test), abdominal ultrasound, and transient elastography in the early detection of MAFLD. **Methods**: An observational prospective study was designed to determine the prevalence of MAFLD and to assess the correlation between complementary tests. Patients were recruited from five health centres. Eligible participants were adults aged between 18 and 70 years with at least one metabolic risk factor, including being overweight (BMI 25–29.9 kg/m^2^) or obese (BMI > 30 kg/m^2^), or diagnosed with type 2 diabetes mellitus (T2DM), dyslipidemia, or metabolic syndrome. The prevalence of MAFLD was calculated. Correlations between diagnostic tests were evaluated using Pearson’s correlation coefficient. **Results**: A total of 98 patients was included. Using CAP (controlled attenuation parameter) measurements, the prevalence of MAFLD was found to be 67.7%, and the prevalence of hepatic fibrosis was 6.5%. The correlation between conventional ultrasound and CAP from FibroScan^®^ for the diagnosis of MAFLD was low and not statistically significant (0.160 [95% CI: −0.100; 0.400], *p* = 0.226). In contrast, the diagnosis of hepatic fibrosis using FibroScan^®^ in PC showed a high correlation with diagnoses performed in gastroenterology department (0.942 [95% CI: 0.844; 0.979], *p* < 0.001). The correlation with biochemical markers was low and not statistically significant for both FIB-4 (0.125 [95% CI: −0.129; 0.363], *p* = 0.334) and the ELF test (0.159 [95% CI: −0.111; 0.407], *p* = 0.246). **Conclusions**: Two out of three patients with metabolic risk factors were diagnosed with MAFLD, while hepatic fibrosis diagnoses were uncommon. These results reinforce the validity of using FibroScan^®^ in PC.

## 1. Introduction

Non-alcoholic fatty liver disease (NAFLD) has become one of the most prevalent hepatic disorders worldwide, and its incidence has steadily increased over the past few decades [1]. It represents the consequence of a metabolic disruption closely associated with insulin resistance and metabolic syndrome [2], and has been widely linked in numerous studies to an increased risk of cardiovascular disease [3,4] and certain neoplastic conditions [5]. Recently, two new terms have been introduced to improve the understanding of the pathophysiology of the disease and to emphasize the underlying metabolic disturbances associated with liver and cardiovascular conditions: metabolic dysfunction-associated fatty liver disease (MAFLD) and metabolic dysfunction-associated steatotic liver disease (MASLD). The diagnostic criteria for MAFLD have been widely validated and shown to be more effective than the previous NAFLD criteria in identifying patients at high risk for both hepatic and extrahepatic complications [6]. More recently, the term MASLD was proposed through a Delphi consensus process, and a new set of diagnostic criteria has been suggested for its identification [7].

The prevalence of MAFLD is unclear, but NAFLD affects approximately 25% of the global population [1], with prevalence rates exceeding 60% among individuals with metabolic risk factors such as obesity, type 2 diabetes mellitus (T2DM), or dyslipidemia [8,9]. Furthermore, MAFLD is associated not only with hepatic complications, including non-alcoholic steatohepatitis (now metabolic dysfunction-associated steatotic liver disease—MASLD), hepatic fibrosis, and cirrhosis, but also with an increased risk of cardiovascular diseases, certain neoplasms, and diabetes-related complications [8,10]. These associations underscore the substantial impact of MAFLD on global morbidity and mortality.

From an economic perspective, MAFLD represents a considerable burden on healthcare systems. In 2016, the annual cost of MAFLD was estimated at €100 billion in the United States and €35 billion across countries such as Germany, Italy, France, and the United Kingdom [11]. These data highlight the need to address MAFLD as a public health priority [12], particularly within the primary care (PC) setting, where most patients with metabolic risk factors are managed.

Although histological confirmation remains the gold standard for diagnosing MAFLD, in clinical practice non-invasive criteria are commonly used to facilitate patient management [6]. Clinical suspicion typically arises from elevated transaminase levels or the presence of suggestive signs of steatosis on abdominal ultrasound, which should be complemented by hepatic fibrosis staging [7], as the degree of fibrosis correlates with long-term mortality. The use of serological markers for screening in PC has been proposed, with subsequent confirmation by elastography at the hospital level [13]. This approach has led to numerous studies in large hospital-based cohorts reporting unexpectedly high prevalence rates, which may be overestimated given that most patients with metabolic risk factors are managed in PC. In this context, the present study was designed with the primary objective of determining the prevalence of MAFLD and hepatic fibrosis in PC. Secondary objectives included characterizing patients according to their metabolic risk factors and assessing the correlation between serological markers (FIB-4, ELF test) abdominal ultrasound, and transient elastography in the early detection of MAFLD.

## 2. Materials and Methods

### 2.1. Study Design

The ESTEATO-GAL study was designed as an observational study with an initial cross-sectional phase to assess the prevalence of MAFLD and the correlation among diagnostic tests, followed by a five-year longitudinal phase to evaluate disease progression. This paper presents the results of the initial cross-sectional phase.

The study, registered under code 2023/045, was approved on 26 January 2023 by the Galician Research Ethics Committee for Medicines (CEIm-G).

### 2.2. Study Population

Patients were recruited from five health centers (Concepción Arenal, Milladoiro, A Estrada, Muros, and Outes) within the Santiago de Compostela Health Area. Eligible participants were adults aged between 18 and 70 years with a high risk of MASLD, i.e., having at least one metabolic risk factor, such as being overweight (BMI 25–29.9 kg/m^2^) or obese (BMI > 30 kg/m^2^), or diagnosed with type 2 diabetes mellitus (T2DM), dyslipidemia, or metabolic syndrome, as defined in the harmonized definition [14]. All patients provided written informed consent to participate in the study.

We estimated a required sample size of 81 patients to determine the prevalence of MAFLD in our population. This calculation was based on an expected prevalence of 30% [1], a precision of 10%, and a 95% confidence interval. Ultimately, 98 patients were recruited, which provided an adequate sample size to address our primary objective.

### 2.3. Variables

Epidemiological data (sex, age, and health center) and relevant medical history (hypertension, T2DM, hypercholesterolemia, obesity, alcohol consumption, smoking, and prior cardiovascular disease) were collected for each participant. The cardiovascular risk of the patients was estimated using SCORE2 [15,16].

Also, peripheral resistance to insulin was estimated using the HOMA index. Insulin resistance (IR) is a critical factor in several metabolic disorders, including type 2 diabetes, obesity, and metabolic dysfunction associated with fatty liver disease (MAFLD) [17]. The HOMA-IR index is calculated using fasting glucose and insulin levels. It is based on the principle that fasting insulin and glucose levels reflect insulin resistance and insulin secretion in the body [17]. It offers the advantage of being more efficient than other indices, it has been validated against the hyperinsulinemic-euglycemic clamp, and is widely used in epidemiological studies [17]. The cutoff points according to laboratory criteria were: <3 (normal resistance), 3–5 (moderate resistance), and >5 (severe resistance) [17].

In addition, diagnostic tests were performed, including calculation of the FIB-4 index, ELF tests, abdominal ultrasound for the diagnosis of hepatic steatosis, and transient elastography with VCTE (vibration-controlled transient elastography). VCTE is a non-invasive technique that measures liver stiffness and, consequently, the degree of fibrosis. The cutoff values used for the 4 levels of disease were 2–7 kPa (F0−F1, normal), 7,5–10 kPa (F2, moderate scarring), 10–14 kPa (F3, severe scarring), and >14 kPa (F4, cirrhosis). The device used in this study also provided CAP (controlled attenuation parameter) measurements, an indicator of the degree of hepatic fat infiltration. The CAP cutoff values for the steatosis grades were <238 dB/m (S0, no steatosis, corresponding to <5% of fat content), 238–259 dB/m (S1, mild steatosis, corresponding to ≥5% of fat content), 260–290 dB/m (S2, moderate steatosis, corresponding to ≥34% of fat content), and >290 dB/m (S3, severe steatosis, corresponding to ≥67% of fat content).

The FIB-4 index (a fibrosis score based on four parameters: patient age, ALT and AST levels, and platelet count) is a validated non-invasive tool for assessing hepatic fibrosis (HF). The FIB-4 index was provided by our reference laboratory and was calculated using the Sterling formula. Patients were stratified into three risk categories based on the FIB-4 results: low risk < 1.3; intermediate risk (≥1.3 to 3.25); and high risk ≥3.25.

The ELF (enhanced liver fibrosis) test is a non-invasive blood test that measures three direct markers of hepatic fibrosis: hyaluronic acid (HA), amino-terminal propeptide of type III procollagen (PIIINP), and tissue inhibitor of metalloproteinase-1 (TIMP-1). Based on the ELF test results, patients were classified into four levels of fibrosis: F1 (portal fibrosis without septa: minimal fibrosis), F2 (portal fibrosis with few septa: moderate or clinically significant fibrosis), F3 (septal fibrosis with numerous septa but no cirrhosis: severe fibrosis), and F4 (cirrhosis).

In this study, the METAVIR scoring system [11] and the Scheuer classification were used to assess the degree of hepatic fibrosis [12].

### 2.4. Statistical Analysis

Descriptive analyses were conducted to characterize the study population using percentages (%) for qualitative variables, and means with standard deviations (SDs) for quantitative variables. The prevalence of MAFLD and hepatic fibrosis was calculated. Associations between variables related to hepatic steatosis were analysed using the chi-square test for qualitative variables, and Student’s *t*-test was used for quantitative variables. For quantitative variables we used parametric statisticals because the Kolmogorov–Smirnov test showed that all variables showed a normal distribution. Correlations between diagnostic tests were evaluated using Pearson’s correlation coefficient, along with 95% confidence intervals (95% CI) and corresponding *p*-values for statistical significance.

A *p*-value < 0.05 was considered statistically significant. All analyses were performed using SPSS software, V27.0 (IBM SPSS Statistics for Windows, Version 27.0. Armonk, NY, USA: IBM Corp).

## 3. Results

### 3.1. Population Characteristics

A total of 98 patients were included in the study. Clinical and epidemiological characteristics are presented in Table 1. Of the participants, 46.9% were women, with a mean age of 61.2 ± 7.3 years. A total of 54.1% were actively employed, while 30.6% were retired. A basic education level was the most common (43.9%). The mean BMI was 30.7 ± 6.4 kg/m^2^, with an obesity prevalence of 55.1% and overweight prevalence of 42.9% Table 1.

Dyslipidemia (59.8%) and sedentary lifestyle (24.7%) were the most prevalent risk factors identified. Metabolic syndrome was present in 47.9% of patients. The mean HOMA index was 2.5 (95% CI: 1.0–3.9), classifying elevated insulin resistance in 13.8% of the patients and moderate insulin resistance in 31.3%.

High cardiovascular risk was identified in 52.8% of patients, and very high risk in 2.2%. A total of 74.5% of patients reported adherence to pharmacological treatment, while 39.8% reported adherence to a Mediterranean-style diet.

### 3.2. Prevalence of MAFLD and Hepatic Fibrosis

The prevalence of MAFLD based on ultrasound findings was 75.3%, whereas CAP measurements indicated a prevalence of 67.7%. According to CAP results, 24.2% of patients had mild steatosis, 14.5% had moderate steatosis, and 29.0% had severe steatosis.

A non-significant trend was observed toward a lower proportion of women among patients with MAFLD (35.7% vs. 55%, *p* = 0.150). No statistically significant differences were observed in age (*p* = 0.252), educational level (*p* = 0.457), employment status (*p* = 0.376), or income level (*p* = 0.715), as shown in Table 2.

The previously observed conditions associated with MAFLD were obesity (59.5% vs. 30.0%, *p* = 0.030) and metabolic syndrome (56.1% vs. 20.0%, *p* = 0.008) (Table 2).

No differences were observed in the blood tests or liver function parameters; however, physical examination revealed higher weight (*p* = 0.001), body mass index (*p* = 0.004), and waist circumference (*p* = 0.007) in patients with steatosis, as shown in Table 3.

In patients with MAFLD, insulin resistance was higher (3.6 [2.1] vs. 1.8 [0.9], *p* < 0.001), and it was commonly classified in the elevated range (57.5% vs. 15.0%, *p* = 0.002). Moreover, 93.5% of the patients did not have hepatic fibrosis (stage F0-F1). However, 37.9% showed some degree of fibrosis according to the FIB-4 index, and 45.3% according to the ELF test.

### 3.3. Correlation Between Different Diagnostic Tests

The correlation between conventional ultrasound and CAP from FibroScan^®^ for the diagnosis of MAFLD was low and not statistically significant (0.160 [95% CI: −0.100; 0.400], *p* = 0.226). The correlation between ultrasound and CAP from FibroScan^®^ for the diagnosis of MASLD was low and not statistically significant (0.095 [95%CI: −0.165, 0.343], *p* = 0.475).

The correlation with CAP measurements performed in gastroenterology departments was slightly higher (0.393 [95% CI: −0.031; 0.820], *p* = 0.261), although this assessment was conducted in only 18 patients.

In contrast, the diagnosis of hepatic fibrosis using FibroScan^®^ in PC showed a high correlation with that performed in gastroenterology departments, both in classification (0.942 [95% CI: 0.844; 0.979], *p* < 0.001) and numerical estimation (0.617 [95% CI: 0.194; 0.847], *p* = 0.008).

The correlation with biochemical markers was low and not statistically significant for both FIB-4 (0.125 [95% CI: −0.129; 0.363], *p* = 0.334) and the ELF test (0.159 [95% CI: −0.111; 0.407], *p* = 0.246).

## 4. Discussion

The findings provide valuable insights into the diagnosis of MAFLD in the PC setting, highlighting that two out of three patients with metabolic risk factors are affected, while hepatic fibrosis remains uncommon. The results underscore the importance of implementing early detection strategies in PC, particularly among patients with metabolic risk factors. This would enable the identification of the disease in its early stages, when it is potentially reversible through timely and effective therapeutic interventions. Moreover, these results reinforce the validity of using FibroScan^®^ in PC, showing strong correlation with assessments conducted in gastroenterology departments, particularly for the evaluation of fibrosis.

To our knowledge, this is the first study to analyze the prevalence of MAFLD in PC settings using the CAP parameter of the Fibroscan^®^ in a sample of patients at high risk of metabolic disorders. Our results demonstrate a high prevalence of MAFLD, affecting two-thirds of the patients. Although the overall prevalence is estimated at 30% [1], it is substantially higher in patients with T2DM, which could reach 60% [18], and in those with obesity, with prevalences of up to 90% [9].

Regarding the validity of serological tests for hepatic fibrosis screening (FIB-4 index and the ELF test), our findings indicate low correlation when compared with transient elastography results. These findings are consistent with those published by the LiverScreen Consortium [19]. To date, the American Diabetes Association (ADA) recommends the use of any of these markers for hepatic fibrosis screening in patients with T2DM [13].

Gracen et al. examined the diagnostic performance of the FIB-4 index, noting that in populations with low hepatic fibrosis prevalence, sensitivity decreases and false negatives increase, thereby limiting the test validity [20]. A systematic review of 11 studies also evaluated the diagnostic performance of the ELF test, concluding that its accuracy is limited in populations with low fibrosis prevalence [21].

Our results, showing a hepatic fibrosis prevalence of 6.5%, confirm that the expected prevalence of hepatic fibrosis in PC is low, despite the high prevalence of MAFLD. This may be explained by the early stage of disease in many patients, who are consequently at low risk of developing fibrosis. This would account for the low correlation observed in our study.

The weak correlation observed between hepatic ultrasound and transient elastography in our study is consistent with previously published evidence. A 2021 review reported that elastography is more sensitive and specific than ultrasound for detecting hepatic steatosis, particularly in the presence of moderate or severe probability of fibrosis. Moreover, steatosis grading by ultrasound is challenging, as it relies on the operator’s subjective visual assessment of fat infiltration [22]. We believe that the questionable correlation between the evaluated biomarkers (FIB-4, ELF test), hepatic ultrasound, and elastography suggests that none of these widely used tests are suitable for the diagnosis of MAFLD in the general population with risk factors. Rather, our data indicate that transient elastography is an effective strategy for the diagnosis and staging of MAFLD in PC, given its high concordance with results obtained by specialists in gastroenterology with extensive experience in this procedure.

The present study provides valuable data on the prevalence and risk factors of MAFLD in PC setting, a field where research remains limited to date. Additionally, the correlation analysis between different diagnostic tests confirms that commonly used tests are not suitable for the diagnosis of steatosis or hepatic fibrosis. However, the findings support the use of elastography in PC, given its strong correlation with results obtained by highly experienced specialists. The current lack of consensus regarding screening guidelines underscores the importance of studies like ours for informing recommendations in future clinical guidelines.

According to previous studies that identified an association between MAFLD and subclinical myocardial dysfunction [23], accurate identification of MAFLD in primary care (PC) could enhance the diagnosis of this dysfunction and support the management of these patients as being at high cardiovascular (CV) risk. Early intervention—through weight reduction, better control of diabetes mellitus, and optimal management of arterial hypertension—has been shown to improve cardiovascular outcomes by reducing CV events. Our results demonstrated that only half of the sample was initially classified as having high or very high CV risk; however, with accurate classification of MAFLD or MASLD, more than half of the patients would fall into a higher CV risk category, which would warrant different clinical management, particularly in PC settings.

Our results reinforce the need to consider MAFLD as a major public health issue, with a high prevalence, particularly among patients with underlying metabolic conditions. These results highlight the importance of a multidisciplinary approach to patient management, addressing not only hepatic pathology but also associated comorbidities, and confirming the complexity of the syndrome. Biochemical markers, such as FIB-4 or ELF, are not reliable for identifying high-risk patients, in contrast with other studies [24,25], with better results with elastography. The use of transient elastography for the diagnosis and staging of MAFLD in our study suggests that it may be a feasible and effective strategy in the PC setting.

A potential limitation of this study is the small sample size, which may have impacted the statistical significance of some results, particularly in the group without MAFLD. Another limitation is the absence of histological confirmation of MAFLD diagnosis. However, the use of validated non-invasive methods largely mitigates this limitation, as they are currently considered the diagnostic reference standard [26]. A major strength of this study is its longitudinal design, which will provide valuable data on the long-term progression of the disease.

## 5. Conclusions

Based on the findings of this study, we conclude that in the PC setting, for patients with risk factors associated with metabolic disorders, the prevalence of MAFLD is high (affecting two-thirds of patients), but the prevalence of hepatic fibrosis is low.

On the other hand, commonly used diagnostic tools for MAFLD, such as abdominal ultrasound and biochemical markers (FIB-4, ELF test) for hepatic fibrosis, showed low correlation with results obtained through transient elastography. Therefore, diagnostic methods with superior accuracy, such as transient elastography, should be recommended.

This study highlights the crucial role of PC in the early detection and management of MAFLD. Our findings reinforce the need for multidisciplinary approaches and preventive strategies tailored to the PC setting. They also underscore the urgent need to develop consensus-based clinical guidelines and to expand access to advanced non-invasive diagnostic tools, such as transient elastography, to support the diagnosis and monitoring of MAFLD, thereby improving disease management.

## Figures and Tables

**Table 1:** Epidemiologic characteristics of the patients in the sample.

	n	Percentage
Women	46	46.9%
Age		61.2 (7.3)
Level of studies		
Basics	43	43.9%
Media	31	31.6%
University students	24	24.5%
Work activity		
Retired	30	30.6%
Unemployed	4	6.1%
Employed	53	54.1%
Domestic Acts	7	7.1%
Student	2	2.0%
Economic income		
TSI 001(Retired < €18,000 per year)	17	17.3%
TSI 002 (Retired between €18,000 and €100,000 per year)	26	26.5%
TSI 003 (Employed < €18,000 per year)	33	33.7%
TSI 004 (Employed between €18,000 and €100,000 per year)	20	20.4%
TSI 005 (>€100,000 per year)	2	2.0%
Personal background		
Type 2 diabetes	30	30.6%
Overweight	42	42.9%
Obesity	54	55.1%
Hypercholesterolemia	58	59.8%
Metabolic Syndrome	46	47.9%
Polycystic Ovary Syndrome	3	3.1%
Preexisting Cardiovascular Disease	6	6.1%
Physical activity		
Low	34	34.7%
Moderate	48	49.0%
Intense	16	16.3%
Smoking		
Smoker	13	13.3%
Ex-smoker	38	38.8%
Alcohol consumption		
None	71	72.4%
1 Standard drink	20	20.4%
2 Standard drinks	4	4.1%
≥3 Standard drinks	3	3.1%

**Table 2:** Epidemiologic characteristics of the patients with hepatic steatosis.

	Hepatic Steatosis	No Steatosis	*p*
Women	35.7%	55.0%	0.150
Age	59.1 (10.8)	61.9 (7.8)	0.252
Level of studies			
Basics	47.6%	50.0%	0.457
Media	33.3%	20.0%	
University students	19.0%	30.0%	
Work activity			
Retired	33.3%	55.0%	0.376
Unemployed	9.5%	0.0%	
Employed	47.6%	40.0%	
Domestic Acts	7.1%	5.0%	
Student	2.4%	0.0%	
Economic income			
TSI 001(Retired < €18,000 per year)	21.4%	30.0%	0.715
TSI 002 (Retired between €18,000 and €100,000 per year)	31.0%	30.0%	
TSI 003 (Employed < €18,000 per year)	21.4%	25.0%	
TSI 004 (Employed between €18,000 and €100,000 per year)	23.8%	10.0%	
TSI 005 (>€100,000 per year)	2.4%	5.0%	
Personal background			
Type 2 diabetes	35.7%	30.0%	0.657
Overweight	40.5%	60.0%	0.150
Obesity	59.5%	30.0%	0.030
Hypercholesterolemia	70.7%	55.0%	0.225
Metabolic Syndrome	56.1%	20.0%	0.008
Polycystic Ovary Syndrome	2.4%	0.0%	0.487
Preexisting cardiovascular disease	7.1%	0.0%	0.220
Physical activity			
Low	31.0%	45.0%	0.550
Moderate	54.8%	45.0%	
Intense	14.3%	10.0%	
Smoking			
Smoker	2.4%	15.0%	0.126
Ex-smoker	45.2%	30.0%	
Alcohol consumption			
None	61.9%	90.0%	0.109
1 Standard drink	28.6%	5.0%	
2 Standard drinks	4.8%	5.0%	
≥3 Standard drinks	4.8%	0.0%	

**Table 3:** Physical examination and blood test results of the patients with hepatic steatosis.

	Hepatic Steatosis	No Steatosis	*p*
Weight (kg)	89.8 (16.5)	75.8 (11.9)	0.001
BMI (kg/m^2^)	31.8 (4.6)	28.4 (3.5)	0.004
Waist circumference (cm)	109.5 (12.6)	100.5 (10.0)	0.007
FIB4	1.4 (0.7)	1.3 (0.5)	0.842
ELF	9.6 (0.7)	9.8 (0.6)	0.266
ALT (UI/mL)	37.1 (30.8)	25.6 (13.6)	0.117
AST (UI/mL)	28.6 (17.5)	24.1 (7.3)	0.278
GGT (UI/mL)	43.1 (18.3)	27.3 (15.5)	0.272
Alkaline phosphatase (UI/mL)	72.6 (23.5)	71.5 (20.0)	0.858
Prothrombin time	10.8 (0.9)	11.2 (0.6)	0.097
Blood glucose	108.0 (25.2)	102.6 (22.7)	0.414
Glomerular rate (mL/min)	94.0 (11.0)	88.3 (13.8)	0.084

BMI: body mass index.

## Data Availability

The data underlying this article are available in RUNA (https://runa.sergas.gal/) and can be accessed at https://10.3390/jcm14093089.org/.

## Abbreviations

The following abbreviations are used in this manuscript:

NAFLD	Non-alcohol fatty liver disease
PC	Primary care
T2DM	Diabetes mellitus type 2
